# Photovoltaic Cell Generations and Current Research Directions for Their Development

**DOI:** 10.3390/ma15165542

**Published:** 2022-08-12

**Authors:** Justyna Pastuszak, Paweł Węgierek

**Affiliations:** Faculty of Electrical Engineering and Computer Science, Lublin University of Technology, Nadbystrzycka 38 A, 20-618 Lublin, Poland

**Keywords:** photovoltaic, solar cells, renewable energy, photovoltaic cell manufacturing technologies, efficiency, photovoltaic generations

## Abstract

The purpose of this paper is to discuss the different generations of photovoltaic cells and current research directions focusing on their development and manufacturing technologies. The introduction describes the importance of photovoltaics in the context of environmental protection, as well as the elimination of fossil sources. It then focuses on presenting the known generations of photovoltaic cells to date, mainly in terms of the achievable solar-to-electric conversion efficiencies, as well as the technology for their manufacture. In particular, the third generation of photovoltaic cells and recent trends in its field, including multi-junction cells and cells with intermediate energy levels in the forbidden band of silicon, are discussed. We also present the latest developments in photovoltaic cell manufacturing technology, using the fourth-generation graphene-based photovoltaic cells as an example. An extensive review of the world literature led us to the conclusion that, despite the appearance of newer types of photovoltaic cells, silicon cells still have the largest market share, and research into ways to improve their efficiency is still relevant.

## 1. Introduction

Concerns about climate change and the increase in demand for electricity due to, among other things, an ever-growing population, necessitate efforts to move away from conventional methods of energy production. Rising carbon dioxide levels in the atmosphere caused by the use of fossil fuels is one of the factors causing ongoing climate change. Switching to renewable energy will produce energy with a smaller environmental footprint compared to fossil fuel sources. We are able to harness the full potential of sunlight energy to develop the best possible energy harvesting technologies capable of converting solar energy into electricity [1].

The currently used solar energy is very marginal—0.015% is used for electricity production, 0.3% for heating, and 11% is used in the natural photosynthesis of biomass. In contrast, about 80–85% of global energy needs are met by fossil fuels. The difficulty with fossil fuels is that their resources are limited and hostile to the environment due to their CO_2_ emissions. For instance, for every ton of coal burned, one ton of carbon dioxide is released into the atmosphere. This emitted carbon dioxide is toxic to the environment and is a primary cause of global warming, the greenhouse effect, climate change, and ozone depletion [2].

The necessity of finding new renewable energy forms is extremely relevant and urgent today. That is why mankind must find alternative sources of energy to provide a clean and sustainable future. Within this context, solar energy is the best option among all alternative renewable energy sources due to its widespread accessibility, universality, and eco-friendly nature [3].

The most common metric used to evaluate the performance of photovoltaic technologies is conversion efficiency, which expresses the ratio of solar energy input to electrical energy output. The efficiency combines multiple component characteristics of the system, such as short-circuit current, open-circuit voltage, and fill factor, which in turn are dependent upon basic material features and manufacturing defects [4].

The cost-effectiveness of making a photovoltaic cell and its efficiency depend on the material from which it is made. Much research in this field has been carried out to find the material that is the most efficient and cost-effective for building photovoltaic cells. The specifications for an ideal material for PV solar cells include the following [5]:

- The cells are expected to have a band gap between 1.1 and 1.7 eV;
- Should have a direct band structure;
- Need to be easily accessible and non-toxic; and
- Should have high photovoltaic conversion efficiency [5].

A key problem in the area of photovoltaic cell development is the development of methods to achieve the highest possible efficiency at the lowest possible production cost. Improving the efficiency of solar cells is possible by using effective ways to reduce the internal losses of the cell. There are three basic types of losses: optical, quantum, and electrical, which have different sources of origin. Reducing losses of any kind requires different, often advanced, methods of cell manufacturing and photovoltaic module production. An upper efficiency limit for commercially accessible technologies is determined by the well-known Shockley–Queisser (SQ) limit, taking into account the balance between photogeneration and radiative recombination [6].

However, the greatest potential lies in the ability to reduce quantum losses, as they are intimately connected with the material properties and internal structure of the cell. Relevant here is the concept of band gap, which defines the minimum required energy of a photon incident onto the cell surface for it to take part in the photovoltaic conversion process. There is a relationship between the efficiency of the cell and the value of the band gap, which in turn is highly dependent on the material from which the photovoltaic cell is made. The basic, commonly used material for solar cells is silicon, which has a band gap value of about 1.12 eV, but by introducing modifications in its crystal structure, the physical properties of the material, especially the band gap width, can be affected [7].

The dominant loss mechanisms in conventional photovoltaic cells are the inability to absorb photons below the band gap and the thermalization of solar photons with energies above the band gap energy. Third-generation solar cell concepts have been proposed to address these two loss mechanisms in an attempt to improve solar cell performance. These solutions aim to exploit the entire spectrum by incorporating novel mechanisms to create new electron–hole pairs [8].

Major development potential among these concepts for improving the power generation efficiency of solar cells made of silicon is shown by the idea of cells whose basic feature is an additional intermediate band in the band gap model of silicon. It is located between the conduction band and the valence band, and its function is to allow the absorption of photons with energies below the width of the energy gap, resulting in higher quantum efficiency (a higher number of excited electrons in relation to the number of photons incident onto the surface of the cell) [9]. Currently, many directions of research development on the introduction of intermediate bands in semiconductors can be identified. One of them is the use of ion implantation, where two methods can be distinguished: introduction of dopants with extremely high concentrations to the substrate of the semiconductor, and implantation of the layer of silicon with high-dose metal ions [10].

**Figure 1:**
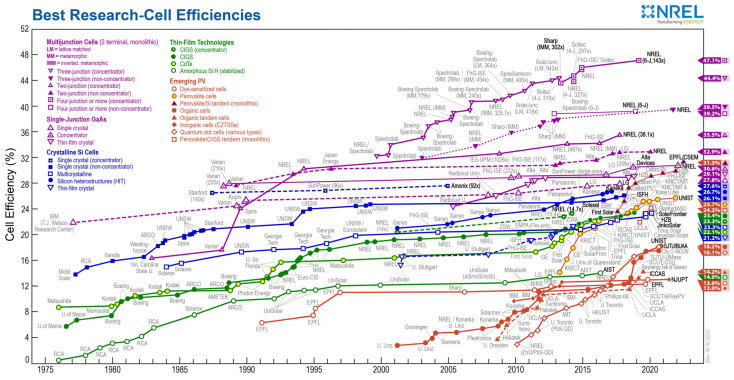
NREL Best Research-Cell Efficiencies chart [11].

The improvement of solar cell efficiency involves reducing various types of losses affecting the resultant cell efficiency. The National Renewable Energy Laboratory (NREL) runs a compilation of the highest verified research cell conversion efficiencies for different photovoltaic technologies, compiled from 1976 to the present (Figure 1). Cell efficiency results are given for each semiconductor family: multi-junction cells; gallium arsenide single-junction cells; crystalline silicon cells; thin film technologies; emerging photovoltaic technologies. The latest world record for an individual technology is indicated by a flag across the right edge containing the efficiency and technology symbol [11].

Photovoltaic cells can be categorized by four main generations: first, second, third, and fourth generation. The details of each are discussed in the next section.

## 2. Photovoltaic Cell Generations

In the past decade, photovoltaics have become a major contributor to the ongoing energy transition. Advances relating to materials and manufacturing methods have had a significant role behind that development. However, there are still numerous challenges before photovoltaics can provide cleaner and low-cost energy. Research in this direction is focused on efficient photovoltaic devices such as multi-junction cells, graphene or intermediate band gap cells, and printable solar cell materials such as quantum dots [12].

**Figure 2:**
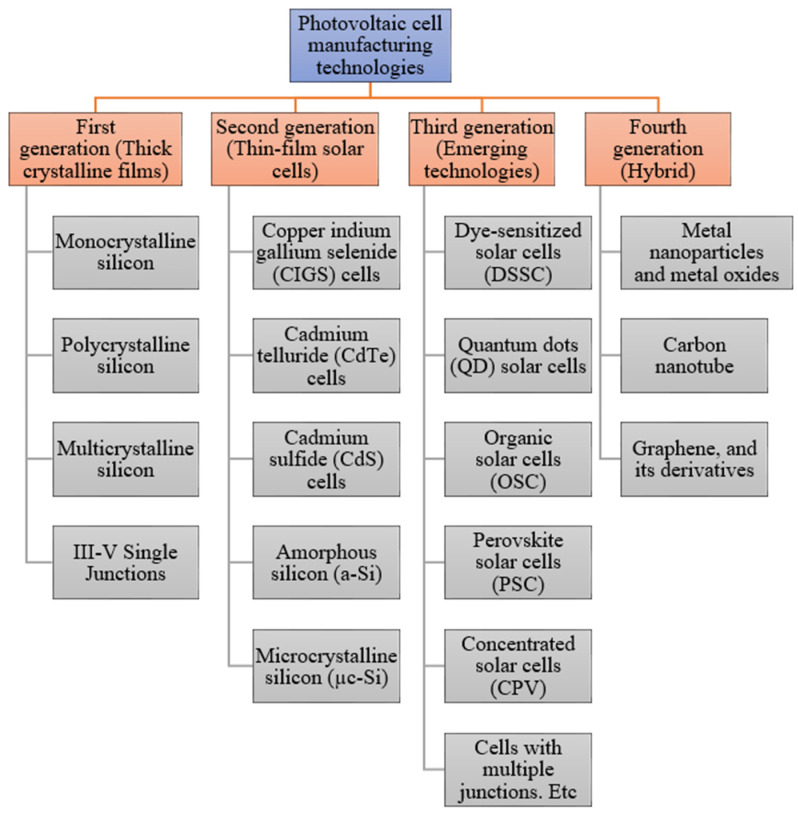
Various solar cell types and current developments within this field [14].

The primary role of a photovoltaic cell is to receive solar radiation as pure light and transform it into electrical energy in a conversion process called the photovoltaic effect. There are several technologies involved with the manufacturing process of photovoltaic cells, using material modification with different photoelectric conversion efficiencies in the cell components. Due to the emergence of many non-conventional manufacturing methods for fabricating functioning solar cells, photovoltaic technologies can be divided into four major generations, which is shown in Figure 2 [13].

The generations of various photovoltaic cells essentially tell the story of the stages of their past evolution. There are four main categories that are described as the generations of photovoltaic technology for the last few decades, since the invention of solar cells [15]:

1. **First Generation:** This category includes photovoltaic cell technologies based on monocrystalline and polycrystalline silicon and gallium arsenide (GaAs).
2. **Second Generation:** This generation includes the development of first-generation photovoltaic cell technology, as well as the development of thin film photovoltaic cell technology from “microcrystalline silicon (µc-Si) and amorphous silicon (a-Si), copper indium gallium selenide (CIGS) and cadmium telluride/cadmium sulfide (CdTe/CdS) photovoltaic cells”.
3. **Third Generation:** This generation counts photovoltaic technologies that are based on more recent chemical compounds. In addition, technologies using nanocrystalline “films,” quantum dots, dye-sensitized solar cells, solar cells based on organic polymers, etc., also belong to this generation.
4. **Fourth Generation:** This generation includes the low flexibility or low cost of thin film polymers along with the durability of “innovative inorganic nanostructures such as metal oxides and metal nanoparticles or organic-based nanomaterials such as graphene, carbon nanotubes and graphene derivatives” [15].

**Figure 3:**
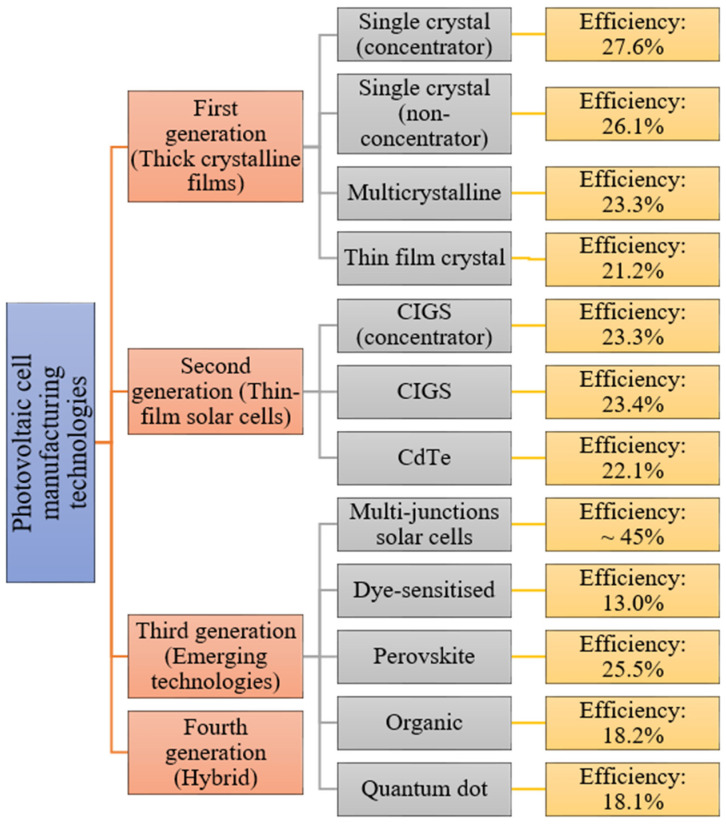
Examples of photovoltaic cell efficiencies [16].

Examples of solar cell types for each generation along with average efficiencies are shown in Figure 3.

### 2.1. First Generation of Photovoltaic Cells

Silicon-based PV cells were the first sector of photovoltaics to enter the market, using processing information and raw materials supplied by the industry of microelectronics. Solar cells based on silicon now comprise more than 80% of the world’s installed capacity and have a 90% market share. Due to their relatively high efficiency, they are the most commonly used cells. The first generation of photovoltaic cells includes materials based on thick crystalline layers composed of Si silicon. This generation is based on mono-, poly-, and multicrystalline silicon, as well as single III-V junctions (GaAs) [17,18].

Comparison of first-generation photovoltaic cells [18]:

- **Solar cells based on monocrystalline silicon (m-si)**

*Efficiency*: 15 ÷ 24%; *Band gap*: ~1.1 eV; *Life span*: 25 years; *Advantages*: Stability, high performance, long service life; *Restrictions*: High manufacturing cost, more temperature sensitivity, absorption problem, material loss.

- **Solar cells based on polycrystalline silicon (p-si)**

*Efficiency*: 10 ÷ 18%; *Band gap*: ~1.7 eV; *Life span*: 14 years; *Advantages*: Manufacturing procedure is simple, profitable, decreases the waste of silicon, higher absorption compared to m-si; *Restrictions*: Lower efficiency, higher temperature sensitivity.

- **Solar cells based on GaAs**

*Efficiency*: 28 ÷ 30%; *Band gap*: ~1.43 eV; *Life span*: 18 years; *Advantages*: High stability, lower temperature sensitivity, better absorption than m-si, high efficiency; *Restrictions*: Extremely expensive [18].

**Figure 4:**
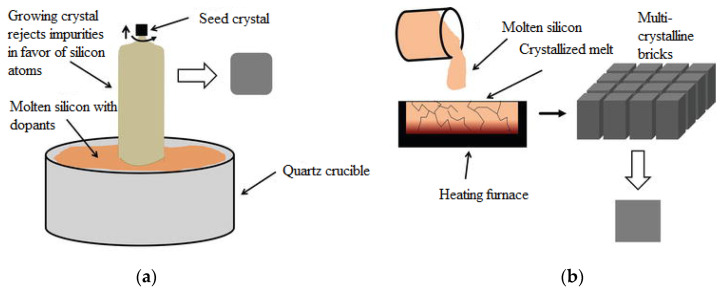
A picture showing (**a**) the Czochralski process for monocrystalline blocks and (**b**) the process of directional solidification for multicrystalline blocks [21].

The first generation concerns p-n junction-based photovoltaic cells, which are mainly represented by mono- or polycrystalline wafer-based silicon photovoltaic cells. Monocrystalline silicon solar cells involve growing Si blocks from small monocrystalline silicon seeds and then cutting them to form monocrystalline silicon wafers, which are fabricated using the Czochralski process (Figure 4a). Monocrystalline material is widely used due to its high efficiency compared to multicrystalline material. Key technological challenges associated with monocrystalline silicon include stringent requirements for material purity, high material consumption during cell production, cell manufacturing processes, and limited module sizes composed of these cells [19].

Multicrystalline silicon blocks are produced through melting high-purity silicon and crystallizing it in a big crucible by directional solidification process (Figure 4b). There is no reference crystal orientation in this process, as in the Czochralski process, and therefore, silicon material with different orientations is produced. The most commonly used base material for solar cells are p-type Si substrates doped with boron. The n-type silicon substrates are also used for the fabrication of high-efficiency solar cells, but they present additional technical challenges, such as achieving uniform doping along the silicon block in comparison to p-type substrates [20].

In the production of crystalline solar cells, six or more steps need to be carried out sequentially. These typically include surface texturing, doping, diffusion, oxide removal, anti-reflective coating, metallization, and firing. At the end of the process, the cell efficiency and other parameters are measured (under standard test conditions). The efficiency of photovoltaic cells is determined by the material quality that is used in their manufacture [21].

The theoretical efficiency threshold for first-generation PV cells appears to have been estimated at 29.4%, and a sufficiently close value was reached as early as two decades ago. At the laboratory scale, reaching 25% efficiency was recorded as early as 1999, and since then, very minimal improvements in efficiency values have been achieved. Since the appearance of crystalline silicon photovoltaic cells, their efficiency has increased by 20.1%, from 6% when they were first discovered to the current record of 26.1% efficiency. There are factors that limit cell efficiency, such as volume defects. Breakthroughs in the production of these cells include the introduction of an aluminum back surface field (Al-BSF) to reduce the recombination rate on the back surface, or the development of Passivated Emitter and Rear Cell (PERC) technology to further reduce the recombination rate on the back surface [22].

#### 2.1.1. Al-BSF Photovoltaic Cells

**Figure 5:**
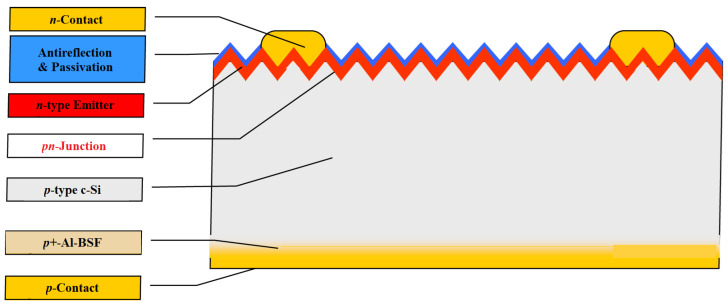
Silicon solar cell structure: Al-BSF [1].

Silicon solar cells with distributed p-n junctions were invented as early as the 1950s, soon after the first semiconductor diodes. Originally, boron diffusion in arsenic-doped wafers was used to form p-n junctions, but now, the industry standard is phosphor diffusion in boron-doped wafers. After the transition in the 1960s from n-type wafers to p-type wafers, the implementation of an aluminum back-surface field (Al-BSF) by fusing the back contact to the substrate made it possible to reduce recombination on the back side (Figure 5). This fairly simple contact screen printing design held a dominant position, with 70–90% of the market share for the past several decades [23].

**Figure 6:**
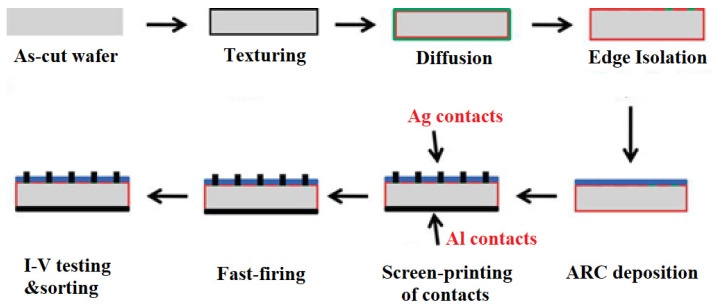
Al-BSF solar cell manufacturing process [21].

Standard aluminum back surface field (Al-BSF) technology is one of the most widely used solar cell technologies due to its relatively simple manufacturing process. It is based on depositing Al entirely on the full rear-side (RS) in a screen-printing process and forming a p+ BSF, which helps repel electrons from the rear-side of the p-type substrate and improves the cell performance. The process flow of Al-BSF solar cell fabrication is shown in Figure 6. Standard commercial solar cell design consists of a front side with a grid and a rear-side with full area contacts [24].

#### 2.1.2. PERC Photovoltaic Cells

**Figure 7:**
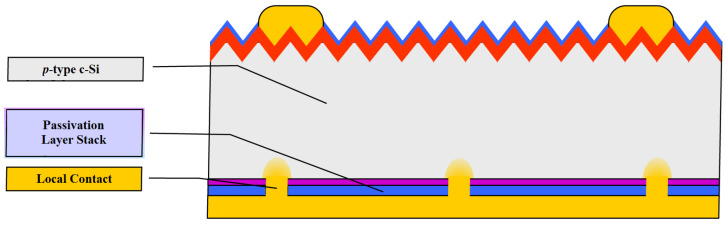
Silicon solar cell structure: PERC [1].

The efficiency of the industrial Al-BSF cell, however, reached about 20% around 2013. It has therefore become attractive to replace the fully contacted Al-BSF cell with a PERC (Passivated Emitter and Rear Cell) structure with local back contacts to achieve enhanced electrical and optical properties (Figure 7). The passivated emitter and rear contact (PERC) solar cell improves the Al-BSF architecture by the addition of a passivation layer on the rear side to improve passivation and internal reflection. Aluminum oxide has been found to be a suitable material for rear side passivation [25].

The capability of this cell structure was demonstrated as early as the 1980s, although it was limited to laboratory processing because of its high cost relative to the yield gain. Moving the PERC technology into mass industrial production in theory involved a comparatively small industry threshold, as only two steps needed to be added to the Al-BSF process, i.e., passivation of the back surface and precise calibration of local back contacts. Nevertheless, decades passed before a profitable PERC process could be developed. A number of reasons led to the implementation of PERC in low-cost, high-volume production, and the increase in productivity to levels ranging from 22% to 23.4% [26]:

- Introduction of aluminum oxide back surface passivation by plasma-enhanced chemical vapor deposition (PECVD) and formation of local back surface field (BSF) by laser ablation of back passivation layer and Al alloy;
- Introduction of a selective emitter process in low-cost manufacturing, a “back-etching” process, or through a laser doping process;
- Reducing the width of front metallization fingers from about 100 μm to less than 30 μm in high-volume production while reducing contact resistance for lightly phosphorus-doped silicon;
- Adding a low-cost hydrogenation step at the end of the cell formation process to passivate volume defects and inactivate boron–oxygen complexes responsible for light-induced degradation (LID); and
- Reappearance of monocrystalline silicon wafers as a result of cost reduction in silicon ingot production by the Czochralski method and the introduction of diamond wire cutting [27].

#### 2.1.3. SHJ-Type Photovoltaic Cells

**Figure 8:**
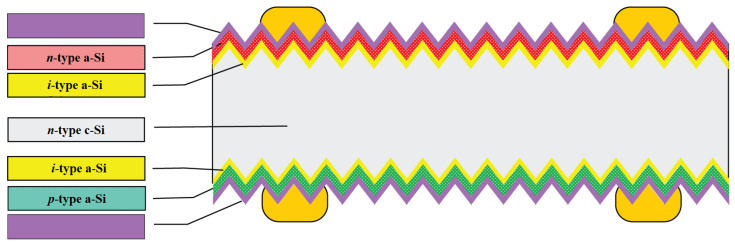
Silicon solar cell structures: heterojunction (SHJ) in rear junction configuration [1].

In parallel with PERC cells, other high-performance cell designs such as interdigitated back contact (IBC) solar cells and heterojunction solar cells (SHJ) have been introduced to mass production. Silicon heterojunction solar cells (SHJ), otherwise referred to as HIT cells, use passivating contacts based on a stack of layers of intrinsic and doped amorphous silicon (Figure 8). Among the major technological challenges associated with this promising cell structure is that once the amorphous silicon layer is deposited, processes above 200 °C cannot be used. This rules out the well-known burned-in screen-printed metal contacts, and thus demands alternative methods using low-temperature pastes or galvanic contacts [28].

There are currently intensive efforts to develop high-capacity production lines that could be competitive with present production standard lines. For SHJ technology to become widespread, there will be a need to overcome the challenges of increased cost of cell manufacturing tools, reducing the use of silver or replacing it with copper by developing Cu electroplating technology, as well as reducing the use of indium in the transparent conductive oxide (TCO) layer [29].

**Figure 9:**
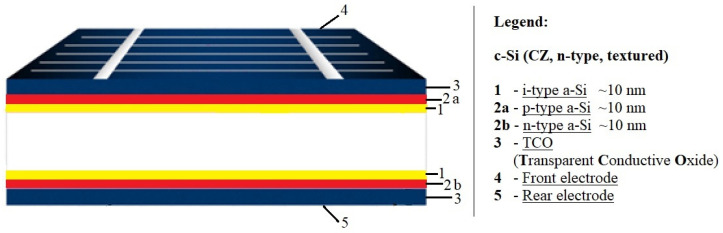
Structure of an HIT solar cell [30].

Moreover, as shown in Figure 9, the HIT solar cell has a symmetric structure, which has two advantages. One is that the cell can be used in what is known as a bifacial module, which can generate more electricity than a regular module, and the other is that the structure is less stressed, which is important when processing thinner wafers [30].

#### 2.1.4. Photovoltaic Cells Based on Single III-V Junctions

GaAs-based single III-V junctions are reviewed at the end of this section. The III-V materials give the greatest photovoltaic conversion efficiency, achieving 29.1% with a GaAs single junction under single sunlight and 47.1% for a six-junction device under concentrated sunlight. These devices are also thinner (absorption layers typically being 2 to 5 µm thick) and thus could be fabricated as lightweight, flexible devices capable of being placed on curved surfaces. The III-V devices have high stability and have a history of high performance for challenging applications such as space [31].

The dominant III-V layer deposition process, metal–organic vapor phase epitaxy (MOVPE), holds the responsibility behind practically every performance record for III-V devices. Yet, historically, this process has been considered as a costly growth technique because of the high cost of precursors, the comparatively low usage of these precursors, and batch growth cycles that require many hours to be completed. Latest studies have significantly improved the growth rate and demonstrated much greater use of precursor chemicals using both MOVPE and hydrogen vapor phase epitaxy (HVPE) techniques, with HVPE also solving the precursor cost problem. Finishing currently includes a great number of labor-intensive, high-priced, and comparatively inefficient process steps, involving photolithography, manual application of spin coating, contact alignment, and metal evaporation and lifting [32].

### 2.2. Second Generation of Photovoltaic Cells

The thin film photovoltaic cells based on CdTe, gallium selenide, and copper (CIGS) or amorphous silicon have been designed to be a lower-cost replacement for crystalline silicon cells. They offer improved mechanical properties that are ideal for flexible applications, but this comes with the risk of reduced efficiency. Whereas the first generation of solar cells was an example of microelectronics, the evolution of thin films required new methods of growing and opened the sector up to other areas, including electrochemistry [33].

The second-generation photovoltaic cell comparison [18]:

- **Solar cells based on amorphous silicon (a-si)**

*Efficiency*: 5 ÷ 12%; *Band gap*: ~1.7 eV; *Life span*: 15 years; *Advantages*: Less expensive, available in large quantities, non-toxic, high absorption coefficient; *Restrictions*: Lower efficiency, difficulty in selecting dopant materials, poor minority carrier lifetime.

- **Solar cells based on cadium telluride/cadium sulfide (CdTe/CdS)**

*Efficiency*: 15 ÷ 16%; *Band gap*: ~1.45 eV; *Life span*: 20 years; *Advantages*: High absorption rate, less material required for production; *Restrictions*: Lower efficiency, Cd being extremely toxic, Te being limited, more temperature-sensitive.

- **Solar cells based on copper indium gallium selenide (CIGS)**

*Efficiency*: 20%; *Band gap*: ~1.7 eV; *Life span*: 12 years; *Advantages*: Less material required for production; *Restrictions*: Very high-priced, not stable, more temperature-sensitive, highly unreliable [18].

#### 2.2.1. CIGS Photovoltaic Cells

A key aspect that needed improvement was reducing the high dependence on semiconductor materials. This was the driving force that led to the emergence of the second generation of thin film photovoltaic cells, which include CIGS. In terms of efficiency, the record value for CIGS is 23.4%, which is comparable to the best silicon cell efficiencies. It should be noted, however, that the efficiency of the research cells does not directly translate to industrially achievable efficiency due to the nature of large-scale processing. Nevertheless, module efficiencies above 20% are already a reality. There has been a significant increase in the efficiency of CIGS cells in recent years and further increases are expected, for example, as a result of further research into alkaline treatment after deposition [34].

Group I-III-VI semiconducting chalcopyrite alloys (Ag,Cu)(In,Ga)(S,Se)_2_, commonly known as CIGS, are particularly favorable absorber materials for solar cells. They have direct band gaps ranging from ~1 to 2.6 eV, high absorption coefficients, and favorable internal defect parameters that allow high minority carrier lifetimes, and solar cells made from them are inherently stable in operation. The first recorded yield was 12% in a monocrystalline device in the mid-1970s. Subsequently, CIGS thin film absorbers, processing, and contacts were greatly improved, resulting in thin film cells with a small area and an efficiency of 23.4%. Current record module efficiencies are 17.6% on glass and 18.6% on flexible steel [35].

CIGS solar cells have been developed in a standard substrate configuration; however, deposition of CIGS at comparatively low temperatures on metal or polymer substrates to form flexible solar products is also possible. CIGS thin films are mainly being deposited by co-evaporation/devaporation or sputtering, and to a minor extent by electrochemical deposition as well as ion beam-assisted deposition. Since these are quaternary compounds, it is critical to control the stoichiometry of the thin film during fabrication. Work is also underway to produce fully or partially solution-deposited CIGS solar cells, and some predict that they could be the ultimate path to ultra-thin, coiled, and flexible PV modules [36].

The steps to improve the efficiency of CIGS cells may be described in the following way: (1) evaporation of CIS compound; (2) reactive elemental bilayer deposition; (3) selenization of sputtered metal precursors; (4) chemical bath deposition of CdS with ZnO:Al as emitter; (5) gallium alloying; (6) sodium alkali incorporation; (7) three-step co-deposition; (8) post-deposition treatment involving heavy alkali ion exchange; and (9) sulfurization after selenization (SAS). Progress is far from linear, with the complete potential for the optimization of the complex interactions between those techniques, along with others under development (e.g., silver alloys), yet to be achieved. A large number of scientists who specialize in CIGS think that efficiencies of 25% can be reached [37].

CIGS is a versatile material that can be produced by many processes and used in a variety of forms. There are currently four main categories of depositing methods used to fabricate CIGS films: (1) metal precursor deposition followed by sulfo-selenization; (2) reactive co-deposition; (3) electrodeposition; and (4) solution processing. All recent world records and the greatest commercial successes have been achieved by two-step sulfo-selenization of metal precursors or reactive co-deposition. CIGS can be deposited on a variety of substrates, including glass, metal films, and polymers. Glass is suitable for making rigid modules, while metal and polymer films allow applications that require lighter or flexible modules. With the evolution of global energy markets toward an appreciation of greenhouse gas reduction and circular economy aspects, the comparatively benign environmental impact of CIGS (especially without CdS) in comparison to different photovoltaic technologies is becoming the next competitive advantage [38].

**Figure 10:**
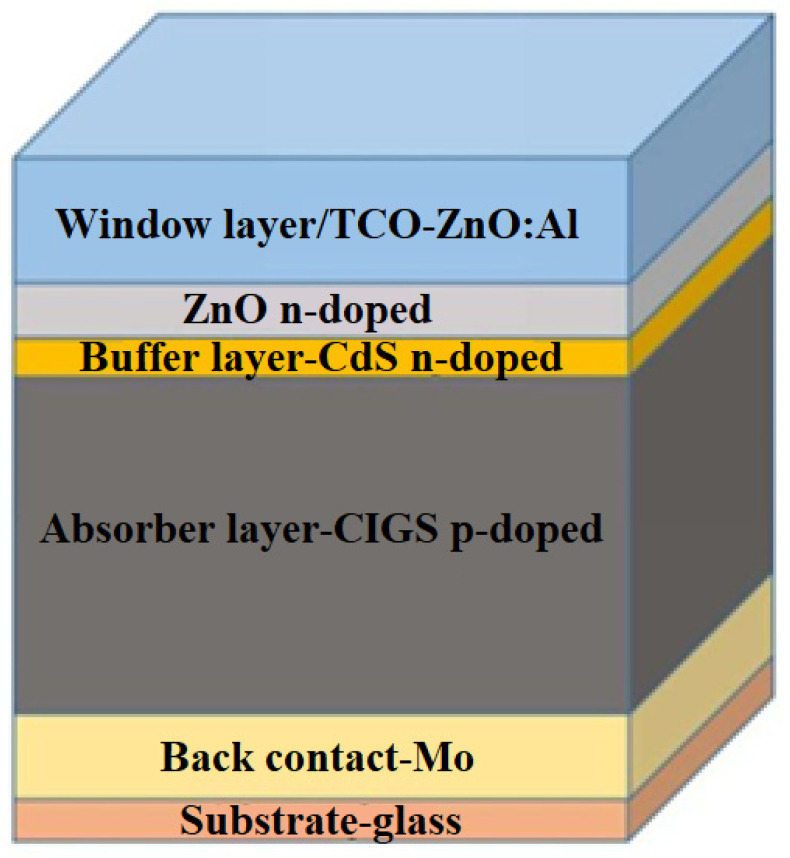
Demonstration of the CIGS-based standard solar cell stack [38].

Photovoltaic cells based on CIGS technology are composed of a pile of thin films deposited on a glass substrate by magnetron sputtering: a bottom molybdenum (Mo) electrode, a CIGS absorbing layer, a CdS buffer layer, and a zinc-doped oxide (ZnO:Al) top electrode. The co-evaporation and CdS buffer layer deposit the CIGS active layer by means of a chemical bath in a regular procedure (Figure 10) [38].

#### 2.2.2. CdTe Photovoltaic Cells

**Figure 11:**
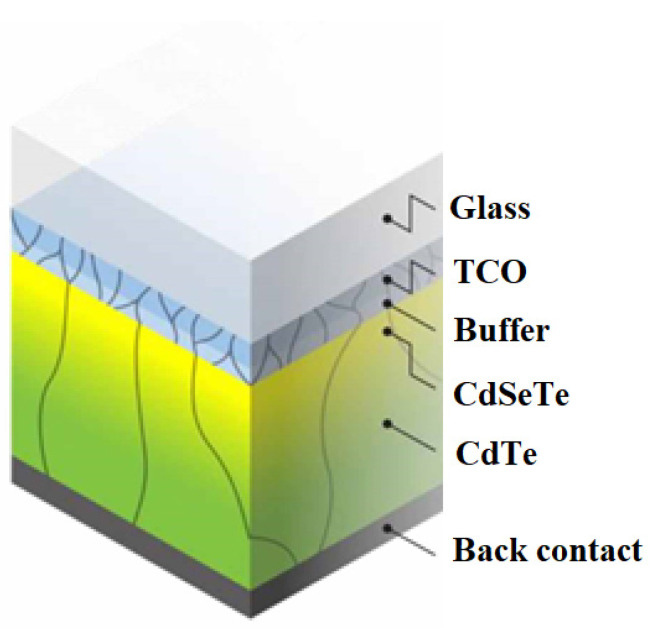
Schematic of a CdTe solar cell [1].

Second-generation photovoltaic cells also include CdTe-based solar cells. An interesting property of CdTe is the reduction in cell size—due to its high spectral efficiency, the absorber thickness can be reduced to about 1 μm without much loss in efficiency, although further work is needed (Figure 11). Super-thin cells are particularly attractive for flexible applications, particularly in building-integrated photovoltaics (BIPV) due to their lighter weight, and transparent photovoltaic panels with CdTe can be developed due to the choice of transparent coating. Their transparency varies from about 10% to 50%, with the disadvantage that an increase in transparency necessarily decreases efficiency. Still, the transparent panels could replace window panels in buildings, not only generating electricity that could be used to power itself, but also contributing to noise reduction and thermal insulation, since most panels are encased in double glass [39].

The technology of CdTe solar cells has developed considerably with the passage of time. In the 1980s, the efficiency of certified cells reached 10%, and in the 1990s, the efficiency was above 15% with the use of a glass/SnO_2_/CdS/CdTe layer structure and annealing in a CdCl_2_ environment, and subsequent Cu diffusion. By the 2000s, efficiency of the cells hit 16.7% using sputtered Cd_2_SnO_4_ and Zn_2_SnO_4_ as transparent conductive oxide (TCO) layers. Over the past decade, new cell efficiency records have reached 22.1%. CdTe technology is increasingly used in rooftop systems and building-integrated photovoltaics [40].

In 2001, NREL produced a cell with an efficiency of 16.5%, which remained the benchmark for about 10 years. The record efficiency has been improved several times in the past 2 years by First Solar and GE Global Research. Currently, CdTe thin films account for less than 10% of the global PV market, with capacity expected to increase. Most of the commercial CdTe cells are manufactured by First Solar, which has achieved record cell efficiencies of 22.1% and average commercial module efficiencies of 17.5–18% [41].

The history of research and development and production of CdTe-based PV cells begins several decades beyond the first studies conducted by Bell Labs (Murray Hill, NJ, USA) in the 1950s on Si crystalline cells. The leading companies have been working on the commercialization of the underlying technology: Matsushita (Kadoma, Osaka, Japan), BP Solar (Madrid, Spain), Solar Cells Inc.—predecessor to First Solar (Tempe, AZ, USA), Abound Solar (Loveland, CO, USA) and GE PrimeStar (Denver, CO, USA). The top manufacturer of thin film CdTe PV is currently First Solar Solar (Tempe, AZ, USA), having fabricated 25 GW of PV modules since 2002 [42].

A range of comparatively easy and inexpensive approaches have been used to produce solar cells with 10–16% efficiency. Examples of several promising cheap deposition techniques include (1) close-space sublimation, (2) spray deposition, (3) electrodeposition, (4) screen printing, and (5) sputtering [43].

Recently, a record efficiency of 16% was reported in a CdS (0.4 μm)/CdTe (3.5 μm) thin film solar cell in which CdS and CdTe layers are deposited using metal–organic CVD (MOCVD) and CSS deposition techniques, respectively. Most of the high-performance solar cells use a device configuration of the superstrate type, where CdTe is deposited on a window layer of CdS. Typically, the structure of the device is composed of glass/CdS/CdTe/Cu-C/Ag. Most of the time, post-deposition heat treatment of the CdTe layer in the presence of CdCl_2_ is necessary to optimize device performance [44].

The recent increase in efficiency is due partly to almost maximum photocurrent by optimizing the optical properties of the cell, deleting parasitically absorbing CdS and introducing CdSe_x_Te_1−x_ with a lower band gap. CdSe_x_Te_1-x_ extends the bandwidth of the absorber from ~1.4 to 1.5 eV and increases the carrier lifetime, thus improving photocurrent collection with no proportional loss of photocurrent. The use of ZnTe in the rear contact also improves the contact ohmicity significantly, and thus the efficiency [45].

#### 2.2.3. Kesterite Photovoltaic Cells

In recent years, kesterite thin film materials have attracted more interest than CdTe and CIGS chalcogenide materials. Cu_2_ZnSnS_x_Se_4−x_ (CZTSSe) thin film photovoltaic material is attracting worldwide attention for its exceptional efficiency and composition derived from the Earth. A lot of research is being conducted on material engineering or designing new architecture to achieve high-performance CZTSSe thin film solar cells. Until recently, the most advanced thin film CZTSSe solar cells have been limited to 11.1% power conversion efficiency (PCE), with these efficiency levels reached using the hydrazine suspension method. Further vacuum and non-vacuum deposition techniques also proved effective in producing CZTSSe solar cells that had a PCE above 8%. Yet still, even record equipment with a PCE of 11% is significantly below the physical limit, generally referred to as the Shockley–Queisser (SQ) limit, which is around 31% efficiency under the Earth’s conditions [46].

A hydrazine-based pure solution method is used to prepare CZTSSe layers, and a Cu-poor and Zn-rich stoichiometry is adopted in the starting solution (Cu/(Zn + Sn) = 0.8 and Zn/Sn = 1.1). Multiple layers of components are spin-coated onto Mo-coated soda-lime glass and annealed at temperatures above 500 °C. Regarding the fabrication of devices, CZTSSe layers are deposited on Mo-coated glass substrates, then 25 nm CdS is deposited in a standard chemical bath and sputtered with 10 nm ZnO/50 nm ITO. A 2 μm thick Ni/Al top metal contact and 110 nm MgF_2_ should be deposited on top of the devices by electron beam evaporation. The area of the device should be determined by mechanical scribing [47].

#### 2.2.4. Photovoltaic Cells Based on Amorphous Silicon

The last type of cells classified as second-generation are devices that use amorphous silicon. Amorphous silicon (a-Si) solar cells are by far the most common thin film technology, whose efficiency is between 5% and 7%, rising to 8–10% for double and triple junction structures. Some varieties of amorphous silicon (a-Si) are amorphous silicon carbide (a-SiC), amorphous germanium silicon (a-SiGe), microcrystalline silicon (μ-Si), and amorphous silicon nitride (a-SiN). Hydrogen is required to dope the material, leading to hydrogenated amorphous silicon (a-Si:H). The gas phase deposition technique is typically used to form a-Si photovoltaic cells with metal or gas as the substrate material [48].

A typical manufacturing process for a-Si:H cells is the roll-to-roll process. First, a cylindrical sheet, usually stainless steel, is rolled out to be used as a deposition surface. The sheet is washed, cut to the desired size, and coated with an insulating layer. Next, a-Si:H is applied to the reflector, after which a transparent conductive oxide (TCO) is deposited on the silicon layer. Finally, laser cuts are made to join the different layers and the module is closed [49].

Amorphous silicon is usually deposited by plasma-enhanced vapor phase deposition (PECVD) at comparatively low substrate temperatures of 150–300 °C. A 300 nm thick a-Si:H layer is capable of absorbing about 90% of photons above the passband in a single pass, allowing the fabrication of lighter and more flexible solar cells [2].

**Figure 12:**
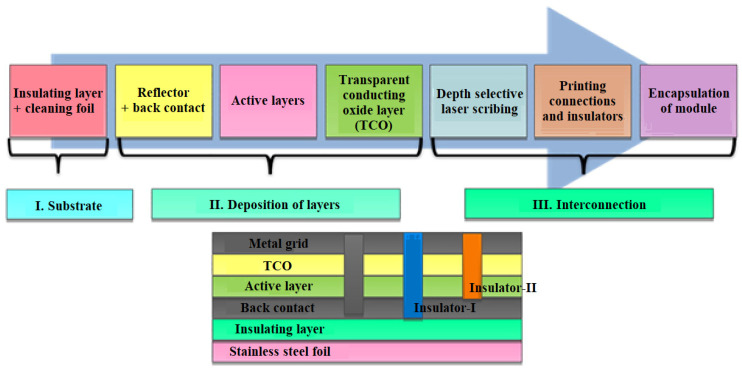
Manufacturing process of a-Si-based solar PV cell [2].

Figure 12 shows the step-by-step fabrication process of an a-Si-based photovoltaic cell. Photovoltaic cells based on thin films are cheaper, thinner, and more flexible compared to first generation photovoltaic cells. The thickness of the light absorbing layer, which was 200–300 µm in first-generation photovoltaic cells, is 10 µm in second-generation cells. Semiconductor materials ranging from “micromorphic and amorphous silicon” to quaternary or binary semiconductors such as “cadmium telluride (CdTe) and copper indium gallium selenide (CIGS)” are used in thin films of photovoltaic cells [50].

### 2.3. Third Generation of Photovoltaic Cells

The third generation of solar cells (including tandem, perovskite, dye-sensitized, organic, and emerging concepts) represent a wide range of approaches, from inexpensive low-efficiency systems (dye-sensitized, organic solar cells) to expensive high-efficiency systems (III-V multi-junction cells) for applications that range from building integration to space applications. Third-generation photovoltaic cells are sometimes referred to as “emerging concepts” because of their poor market penetration, even though some of these have been studied for more than 25 years [51].

The latest trends in silicon photovoltaic cell development are methods involving the generation of additional levels of energy in the semiconductor’s band structure. The most advanced studies of manufacturing technology and efficiency improvements are now concentrated on third-generation solar cells.

One of the current methods to increase the efficiency of PV cells is the introduction of additional energy levels in the semiconductor’s band gap (IBSC and IPV cells) and the increasing use of ion implantation in the manufacturing process. Other innovative third-generation cells that are lesser-known commercial “emerging” technologies include [52]:

1. Organic materials (OSC) photovoltaic cells;
2. Perovskites (PSC) photovoltaic cells;
3. Dye-sensitized (DSSC) photovoltaic cells;
4. Quantum dots (QD) photovoltaic cells; and
5. Multi-junction photovoltaic cells [52].

Third-generation photovoltaic cell comparison [18]:

- **Solar cells based on dye-sensitized photovoltaic cells**

*Efficiency*: 5 ÷ 20%; *Advantages*: Lower cost, low light and wider angle operation, lower internal temperature operation, robustness, and extended lifetime; *Restrictions*: Problems with temperature stability, poisonous and volatile substances.

- **Solar cells based on quantum dots**

*Efficiency*: 11 ÷ 17%; *Advantages*: Low production cost, low energy consumption; *Restrictions*: High toxicity in nature, degradation.

- **Solar cells based on organic and polymeric photovoltaic cells**

*Efficiency*: 9 ÷ 11%; *Advantages*: Low processing cost, lighter weight, flexibility, thermal stability; *Restrictions*: Low efficiency.

- **Solar cells based on perovskite**

*Efficiency*: 21%; *Advantages*: Low-cost and simplified structure, light weight, flexibility, high efficiency, low manufacturing cost; *Restrictions*: Unstable.

- **Multi-junction solar cells**

*Efficiency*: 36% and higher; *Advantages*: High performance; *Restrictions*: Complex, expensive [18].

#### 2.3.1. Organic and Polymeric Materials Photovoltaic Cells (OSC)

Organic solar cells (OSCs) are beneficial in applications related to solar energy since they have the potential to be used in a variety of prospects on the basis of the unique benefits of organic semiconductors, including their ability to be processed in solution, light weight, low cost, flexibility, semi-transparency, and applicability to large-scale roll-to-roll processing. Solution-processed organic solar cells (OSCs) that absorb near-infrared (NIR) radiation have been studied worldwide for their potential to be donor:acceptor bulk heterojunction (BHJ) compounds. In addition, NIR-absorbing OSCs have attracted attention as high-end equipment in next-generation optoelectronic devices, such as translucent solar cells and NIR photodetectors, because of their potential for industrial applications. With the introduction of non-fullerene acceptors (NFAs) that absorb light in the NIR range, the value of OSC is increasing, while organic donor materials capable of absorbing light in the NIR range have not yet been actively studied compared to acceptor materials that absorb light in the NIR range [53].

The most advanced BHJ structure by combining organic donor and acceptor materials showed tremendous hope for low-cost and lightweight organic solar cells. Over the past decade, enormous progress was made, with power conversion efficiencies reaching more than 14% for a single-junction device and more than 17% for a tandem device through the design of new NIR photoactive materials with low bandwidth. Compared to wide-band organic photovoltaic materials, low-band donor and non-fullerene acceptor materials with wide-range solar coverage extended to the NIR region typically exhibit more tightly superimposed electronic orbitals, easier delocalization of π electrons, higher dielectric constant, stronger dipole moment, and lower exciton binding energy. These properties make low-bandwidth photovoltaic materials play an important role in high-performance organic solar cells, including single-junction and tandem devices [54].

A clever strategy in active layer design could be summed up as optimizing the weight ratio of donor to acceptor materials, using ultra-low band gap materials as a third component to improve NIR light utilization efficiency, and adjusting the thickness of the active layer to achieve a compromise between photon collection and charge accumulation. Much effort has gone into optimizing the translucent top electrode: well-balanced conductivity and transmittance in the visible light range, increased reflectance in the NIR or ultraviolet (UV) light range, and better compatibility with active layers. In terms of device engineering, photon crystal, anti-reflection coating, optical microcavity, and dielectric/metal/dielectric (DMD) structures have been placed to realize selective transmission and reflection for simultaneous improvement of power conversion efficiency and average transmission of translucent OSC visible light [55].

#### 2.3.2. Dye-Sensitized Photovoltaic Cells (DSSC)

**Figure 13:**
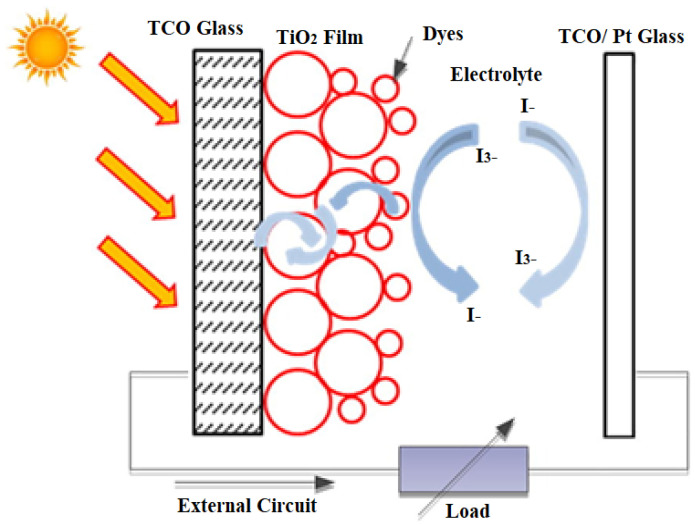
Schematic representation of a DSSCs [2].

Conjugated polymers and organic semiconductors have been successful in flat panel displays and LEDs, so they are considered advanced materials in the current generation of photovoltaic cells. A schematic representation of dye-sensitized organic photovoltaic cells (DSSCs) is shown in Figure 13. Polymer/organic photovoltaic cells can also be divided into dye-sensitized organic photovoltaic cells (DSSCs), photoelectrochemical photovoltaic cells, and plastic (polymer) and organic photovoltaic devices (OPVDs), differing in mechanism of operation [56].

Dye-sensitized solar cells (DSSCs) represent one of the best nanotechnology materials for energy harvesting in photovoltaic technologies. It is a hybrid organic–inorganic structure where a highly porous, nanocrystalline layer of titanium dioxide (TiO_2_) is used as a conductor of electrons in contact with an electrolyte solution also containing organic dyes that absorb light near the interfaces. A charge transfer occurs at the interface, resulting in the transport of holes in the electrolyte. The power conversion efficiency has been shown to be about 11%, and commercialization of dye-sensitized photovoltaic modules is underway. A novel feature in DSSC solar cells is the photosensitization of nanosized TiO_2_ coatings in combination with optically active dyes, which increases their efficiency to more than 10% [57].

DSSCs hold promise as photovoltaic devices because of their simple fabrication, low material costs, and their benefits in transparence, color capability, and mechanical flexibility. The main challenges in commercializing DSSCs are poor photoelectric conversion efficiency and cell stability. The highest attainable theoretical energy conversion efficiency was estimated at 32% for DSSCs; however, the highest efficiency reported to date is only 13%. Intensive work is underway to understand the parameters governing the DSSC to improve its efficiency. Numerous attempts have been made to optimize the redox pair and absorbance of the dye, modify a wide band gap semiconductor as a working electrode, and develop a counter electrode (CE). In addition to increasing the efficiency of DSSC, the cost of materials is another major issue that needs to be solved in future work [58].

#### 2.3.3. Perovskite Photovoltaic Cells

Perovskite solar cells (PSCs) are a revolutionary new photovoltaic cell concept that relies on metal halide perovskites (MHPs), e.g., methylammonium iodide as well as formamidine lead iodide (MAPbI_3_ or FAPbI_3_, respectively). MHPs integrate a number of features favored in photovoltaic absorbers, including a direct band gap with a high absorption coefficient, long carrier lifetime and diffusion length, low defect density, and ease of tuning the composition and band gap. In the year 2009, MHP was first described as a sensitizer in a dye cell based on liquid electrolyte conducting holes. In 2012, MHP demonstrating ~10% efficiency of PSCs based on a solid-state hole conductor sparked an explosion of PSC studies. In about a decade of research, the efficiency of a single PSC junction increased to a certified level of 25.2% [59].

The development of PSCs has been heavily influenced by the improvement of material quality through a broad range of synthetic methods designed under the guidance of a fundamental understanding of MHP growth mechanisms. Comprehension of the complex and correlated processes of perovskite growth (e.g., nucleation, grain growth, as well as microstructure evolution) has aided in the development of a broad range of high-efficiency growth modes (for example, single-step growth, sequential growth, dissolution process, vapor process, post-deposition processing, non-stoichiometric growth, additive-assisted growth, and fine-tuning of structure dimensions). The latest efforts were concentrated on interface engineering, focusing on reducing open-circuit voltage losses and improving stability, particularly by introducing a two-dimensional perovskite surface layer. With progress in synthetic control, the perovskite composition is becoming simpler, mainly toward FAPbI_3_. This will undoubtedly contribute to the simplification of scale deposition methods and a basic understanding of the properties of these cells [60].

#### 2.3.4. Quantum Dots Photovoltaic Cells

**Figure 14:**
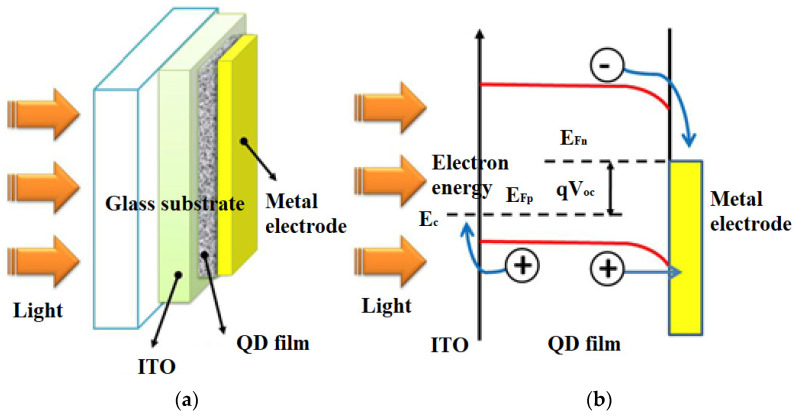
(**a**) A scheme of a solar cell based on quantum dots, (**b**) solar cell band diagram [64].

Solar cells made from these materials are called quantum dots (QDs) and are also known as nanocrystalline solar cells. They are fabricated by epitaxial growth on a substrate crystal. Quantum dots are surrounded by high potential barriers in a three-dimensional shape, and the electrons and electron holes in a quantum dot become discrete energy because they are confined in a small space (Figure 14). Consequently, the ground state energy of electrons and electron holes in a quantum dot depends on the size of the quantum dot [61].

Nanocrystalline cells have relatively high absorption coefficients. Four consecutive processes occur in a solar cell: (1) light absorption and exciton formation, (2) exciton diffusion, (3) charge separation, and (4) charge transport. Due to the poor mobility and short lifetime of excitons in conducting polymers, organic compounds are characterized by small exciton diffusion lengths (10–20 nm). In other words, excitons that form far from the electrode or carrier transport layer recombine and the conversion efficiency drops [62].

The development of thin film solar cells with metal halide perovskites has led to intensive attention to the corresponding nanocrystals (NCs) or quantum dots (QDs). Today, the record efficiency of QD solar cells was improved to 16.6% using mixed colloidal QDs with perovskites. The universality of these new nanomaterials regarding ease of fabrication and the ability to tune the band gap and control the surface chemistry allows a variety of possibilities for photovoltaics, such as single-junction, elastic, translucent, controlled cells with heterostructures and multi-junction tandem solar cells which would push the field even further. However, a narrower size distribution has the potential to enhance the performance of QD solar cells through more ways than one. Firstly, electron transport might be better in smaller QDs, as larger QDs function as a band tail or shallow trap that makes transport more difficult. Secondly, the open-circuit voltage (V_OC_) of QD solar cells could be limited by the smallest band gap (largest size) QD near the contacts. Enhancing the homogeneity and uniformity of QD size would also improve PV performance by the minimization of such losses. Although controlled experiments such as these have not yet been reported, it is possible that more controlled synthesis might provide benefits to QD cells [63].

#### 2.3.5. Multi-Junction Photovoltaic Cells

Multi-junction (MJ) solar cells consist of plural p-n junctions fabricated from various semiconductor materials, with each junction producing an electric current in response to light of a different wavelength, thereby improving the conversion of incident sunlight into electricity and the efficiency of the device. The concept to use various materials with different band gaps has been suggested to utilize the maximum possible number of photons and is known as a tandem solar cell. An entire cell could be fabricated from the same or different materials, giving a broad spectrum of possible designs [65].

**Figure 15:**
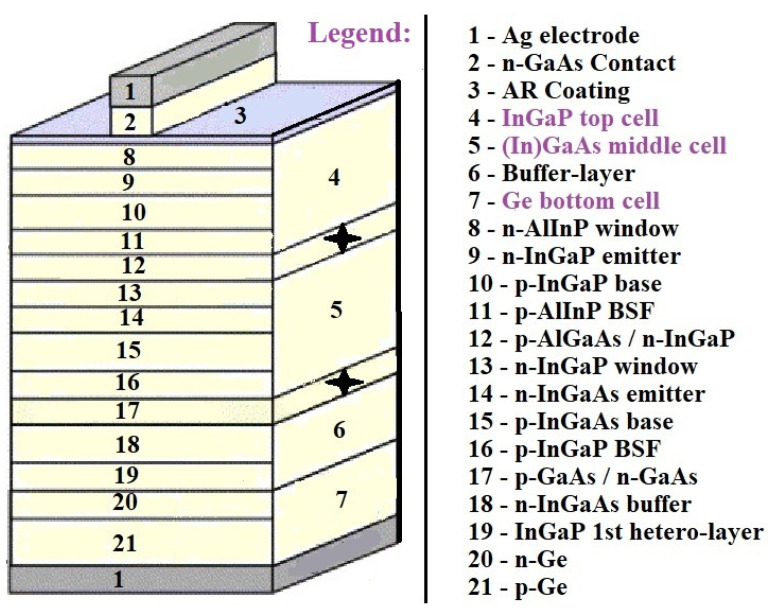
Schematic illustration of a triple-junction cell and approaches for improving efficiency of the cell [65].

Usually, the cells are integrated monolithically and connected in series through a tunnel junction, and current matching between cells is obtained through adjusting each cell’s band gap and thickness. The theoretical feasibility of using multiple band gaps was examined and was found to be 44% for two band gaps, 49% for three band gaps, 54% for four band gaps, and 66% for an infinite number of gaps. Figure 15 illustrates a scheme of an InGaP/(In)GaAs/Ge triple solar cell and presents crucial technologies to enhance efficiency of conversion [66].

Grid-matched InGaP/(In)GaAs/Ge triple solar cells have been widely used in space photovoltaics and have achieved the highest true efficiency of over 36%. Heavy radiation bombardment of various energetic particles in the space environment inevitably damages solar cells and causes the formation of additional non-radiative recombination centers, which reduces the diffusion length of minority carriers and leads to a reduction in solar cell efficiency. The sub-cells in multi-junction solar cells are connected in series; the sub-cell with the greatest radiation degradation degrades the efficiency of the multi-junction solar cell. To improve the radiation resistance of (In)GaAs sub-cells, measures such as reducing the dopant concentration, decreasing the thickness of the base region, etc., can be used [66].

#### 2.3.6. Photovoltaic Cells with Additional Intermediate Band

The National Renewable Energy Laboratory (NREL) estimates that multi-junction and IBSC photovoltaic cells have the highest efficiency under experimental conditions (47.1%). The main feature of these cells is precisely the additional intermediate band in the band gap of silicon. Currently, two types of these cells are specified in the world literature: IBSC (Intermediate Band Solar Cells) and IPV (Impurity Photovoltaic Effect) [67].

Impurity Photovoltaic Effect (IPV) is one of the solutions used to increase the infrared response of PV cells and thus increase the solar-to-electric energy conversion efficiency. The idea of the IPV effect is based on the introduction of deep radiation defects in the structure of the semiconductor crystal structure. These defects ensure a multi-step absorption mechanism for photons with energies below the band gap width. The addition of IPV dopants into silicon solar cell structure, under certain conditions, increases the spectral response, short circuit current density, and conversion efficiency [68].

**Figure 16:**
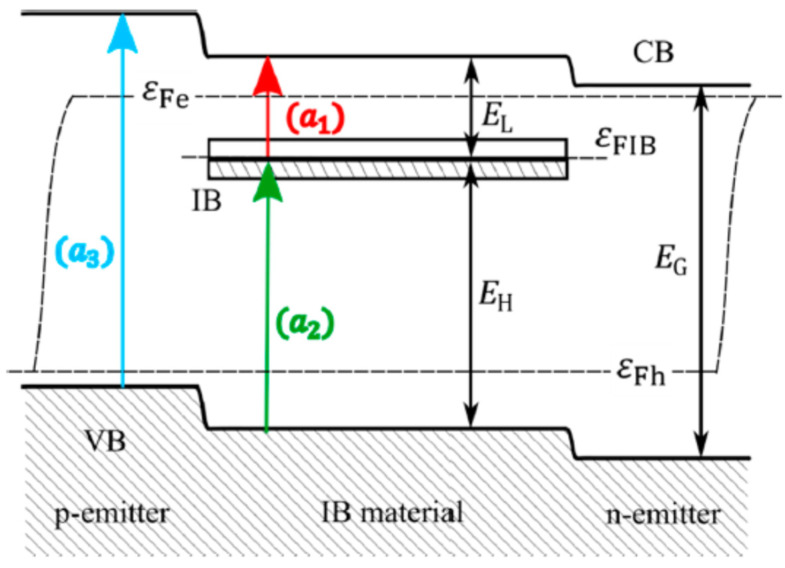
Energy band diagram of an intermediate band solar cell (IBSC) [69].

A major direction of study with great potential for development is Intermediate Band Solar Cells (IBSCs). They represent a third-generation solar cell concept and involve not only silicon, but also other materials. The idea behind the intermediate band gap solar cell (IBSC) concept is to absorb photons with an energy corresponding to the sub-band width in the cell structure. These photons are absorbed by a semiconductor-like material that, in addition to the conduction and valence bands, has an intermediate band (IB) in the conventional semiconductor’s band gap (Figure 16). In IBSCs, the silicon layers are implanted with very high doses of metal ions to create an additional energy level [69].

Based on the research conducted on the effect of defects introduced into the silicon structure, a model was developed according to which introducing selected deep defects into the charge carrier capture region results in improved PV cell efficiency. Of particular interest are defects that facilitate the transport of majority carriers and defects that counteract the accumulation of minority carriers. This contributes significantly to reducing the recombination process at the charge carrier capture site. Finally, by introducing defects into the structure of the silicon underlying the solar cell, we combine effective surface passivation with simultaneous reduction in optical losses [70].

The introduction of intermediate bands in semiconductors, using ion implantation, can be executed using two methods: by introducing dopants of very high concentration into the semiconductor substrate, or by implanting the silicon layer with high-dose metal ions. The increasing use of ion implantation in the photovoltaic cell manufacturing process has the potential to reduce the cost of deployment and increase the cost-effectiveness of silicon cells by increasing their efficiency. The use of ion implantation technology provides increased precision of silicon layer doping and generation of additional levels of energy in the band gap, as well as shortening the individual stages of cell fabrication, which ultimately translates into improved quality and lower production costs [71].

**Figure 17:**
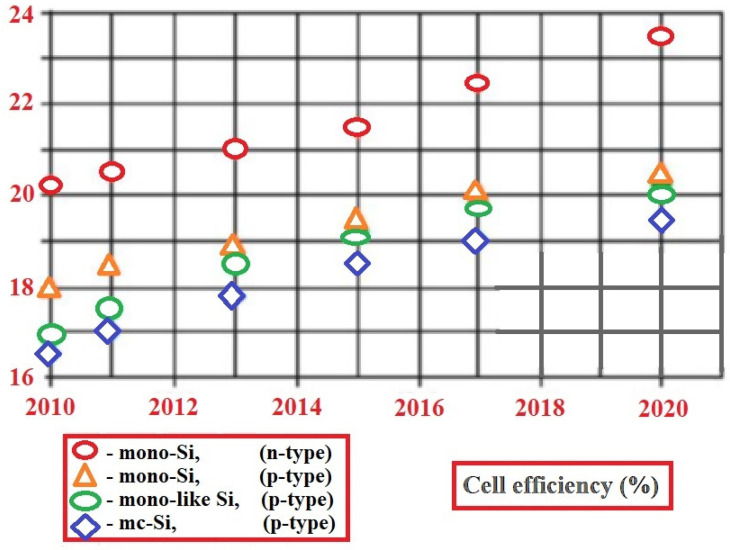
Stabilized cell efficiency trend curves [72].

Lately, the technique of ion implantation is gaining popularity in the solar industry, gradually displacing the diffusion technique that has been used for many years. As can be seen in Figure 17, cell performance is expected to continue to improve as the technology evolves toward higher efficiencies. In addition to local and reference doping, the major benefits of this technology involve high precision control of the amount and distribution of dopant doses, which results in high uniformity, repeatability, and increased efficiency (above 19%), with a significantly narrower distribution of cell performance [72].

In the method of ion implantation, chosen ions with the required impurity are inserted into the semiconductor by accelerating the impurity ions to a high energy level and implanting the ions into the semiconductor. The energy given to the impurity ions defines the depth of ion implantation. Contrary to the diffusion technology (where the impurity ion dose is introduced only at the surface), in the ion implantation technique, a controllable dose of impurity ions can be placed deeply into the semiconductor [73].

### 2.4. Fourth Generation of Photovoltaic Cells

Fourth-generation photovoltaic cells are also known as hybrid inorganic cells because they combine the low cost and flexibility of polymer thin films, with the stability of organic nanostructures such as metal nanoparticles and metal oxides, carbon nanotubes, graphene, and their derivatives. These devices, often referred to as “nanophotovoltaics”, could become the promising future of photovoltaics [74].

#### Graphene-Based Photovoltaic Cells

By using thin polymer layers and metal nanoparticles, as well as various metal oxides, carbon nanotubes, graphene, and their derivatives, the fourth generation provides excellent affordability and flexibility. Particular emphasis was placed on graphene because it is considered a nanomaterial of the future. Due to their unique properties, such as high carrier mobility, low resistivity and transmittance, and 2D lattice packing, graphene-based materials are being considered for use in PV devices instead of existing conventional materials. However, to achieve adequate device performance, the key to its practical applications is the synthesis of graphene materials with appropriate structure and properties [75].

Since the properties of graphene are fundamentally related to its fabrication process, a judicious choice of methods is essential for targeted applications. In particular, highly conductive graphene is suitable for use in flexible photovoltaic devices, and its high compatibility with metal oxides, metallic compounds, and conductive polymers makes it suitable for use as a selective charge-taking element and electrode interlayer material [76].

In the past two decades, graphene has been combined with the concept of photovoltaic material and is showing a significant role as a transparent electrode, hole/electron transport material, and interfacial buffer layer in solar cell devices. We can distinguish several types of graphene-based solar cells, including organic bulk heterojunction (BHJ) cells, dye-sensitized cells, and perovskite cells. The energy conversion efficiency exceeded 20.3% for graphene-based perovskite solar cells and reached 10% for BHJ organic solar cells. In addition to its function of extracting and transporting charge to the electrodes, graphene plays another unique role—it protects the device from environmental degradation through its packed 2D lattice structure and ensures the long-term environmental stability of photovoltaic devices [77].

**Figure 18:**
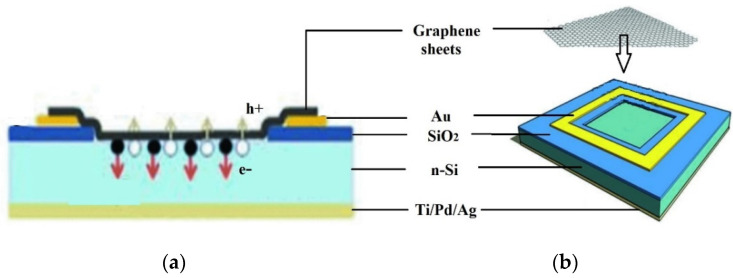
Graphene–silicon Schottky junction solar cell. (**a**) Cross-sectional view, (**b**) schematic illustration of the device configuration [75].

Semi-metallic graphene having a zero band gap creates Schottky junction solar cells with silicon semiconductors. Even though graphene was discovered for the first time in 2004, the first graphene–silicon solar cell was not characterized as an n-silicon cell until 2010. Figure 18 schematically shows a graphene–silicon solar cell with a Schottky junction. Graphene sheets (GS), cultured by chemical vapor deposition (CVD) on nickel films, were wet deposited on pre-patterned Si/SiO_2_ substrates with an effective area of 0.1–0.5 cm^2^. The graphene sheet forms a coating on the exposed n-Si substrate, creating a Schottky junction. The graphene sheet was contacted using Au electrodes [78].

Graphene synthesis uses mainly two methodologies, which are the bottom-up and top-down methods. In the top-down approach, graphite is the starting material, and the goal is to intercalate and exfoliate it into graphene sheets by solid, liquid, or electrochemical exfoliation. Another approach under this categorization is the exfoliation of graphite oxide into graphene oxide (GO), after which chemical or thermal reduction takes place. A bottom-up approach is to produce graphene from molecular precursors by chemical vapor deposition (CVD) or epitaxial growth. The structure, morphology, and attributes of the resulting graphene, including the layer numbers, level of defects, electrical and thermal conductivity, solubility, and hydrophilicity or hydrophobicity, are dependent on the manufacturing process [78,79].

Graphene can absorb 2.3% of incident white light even though it is only one atom thick. Incorporating graphene into a silicon solar cell is a promising platform since graphene has a strong interaction with light, fulfilling both the optical (high transmittance) and electrical (low layer resistance) requirements of a typical transparent conductive electrode. It is important to note that both the layer resistance and the transmittance of graphene change with the number of layers. As the layer resistance decreases as the number of graphene layers increases, the optical transparency decreases as well [80].

For PV technology, graphene offers a lot more because of its flexibility, environmental stability, low electrical resistivity, and photocatalytic features, while having to be carefully and deliberately designed for the targeted applications and specific requirements [78,80].

One problem for graphene application is the absence of a simpler, more reliable way to deposit a well-ordered monolayer with low-cost flakes on target substrates having various surface properties. The other problem is the adhesion of the deposited graphene thin film, a subject that has not yet been studied properly. Large-area continuous graphene layers with high optical transparency and electrical conductivity may be fabricated by CVD. As an anode in organic photovoltaic devices, graphene holds great promise as a replacement for indium tin oxide (ITO) because of its inherently low-cost manufacturing process and excellent conductivity and transparency properties [81].

Graphene’s major disadvantage is its poor hydrophilicity, which negatively affects the design of devices processed in solution, but that fact may be overcome through modifying the surface by non-covalent chemical functionalization. Given graphene’s mechanical strength and flexibility, as well as its excellent conductivity properties, it can be anticipated that new applications in plastic electronics and optoelectronics will soon emerge involving this new class of CVD graphene materials. The discovery paves the way for low-cost graphene layers to replace ITO in photovoltaic and electroluminescent devices [82].

## 3. Prospects and Research Directions

Since the beginning of photovoltaic cells, crystalline silicon-based photovoltaic technology has played a dominant role in the market, with crystalline PV modules accounting for about 90% of the market share in 2020. In recent years, there has been a rapid development of thin film solar cells (such as cadmium telluride (CdTe) and indium–gallium selenium compounds (CIGS) cells) and new solar cells (such as dye-sensitized solar cells (DSSCs), perovskite solar cells (PSCs), quantum dot solar cells (QDSCs), etc.) [83].

The growing interest in BIPV systems has contributed to the overall development of photovoltaic technology, which has led to lower costs, increasing the feasibility of investment. Most of the standard second-generation technologies show efficiencies of 20–25%, and while they are expensive, the cost of silicon cells has come down and it is the improvement of silicon technologies that is now one of the key research directions [84].

Graphene and its derivatives are a promising area of research as they are in the early stages of research and development. The goal of using carbon nanostructures is to produce energy-efficient products that combine transport, active, and electrode layers. Many researchers in contemporary graphene research are now focusing on new graphene derivatives and their novel applications in manufacturing devices [85].

Nevertheless, the technologies used for third- and fourth-generation cells are still in the prototyping stage. Production-scale prototypes have also been built and have been successful (10–17% efficiency). In contrast, third-generation multi-junction cells are already commercially available and have achieved exceptional conversion factors (from 40% to over 50%) that place this alternative as the best [85]. Considering the market trends of increasing use of intermediate energy levels in PV cell production, it makes perfect sense to conduct research in this direction, which is exactly what our research team is doing.

The practical realization of the idea of energy-efficient IBSC-type silicon solar cells with intermediate energy levels in the band gap of the semiconductor, produced by ion implantation, needs more studies directed at the search for the optimal implantation parameters, which is the energy, type, and dose of ions, adjusted to the substrate material properties, particularly the level and type of dopant [86].

It appears that implantation can also lead to a reduction in the optical losses present in the cell. Impurities and defects introduced into the silicon crystal lattice under the right conditions can create additional intermediate band gaps, which realistically contributes to the reduction in the energy gap width. As a result, some photons with energies lower than the band gap value cause the formation of additional electron–hole pairs. The existence of this additional energy band contributes to the increase in the value of the photoelectric current, which results from the absorption of photons not previously involved in the photovoltaic conversion process. The range of absorbed light radiation increases toward the infrared, and after absorbing a photon from this range, the electron goes first to the intermediate band and then to the conduction band [87].

Our long-standing studies on changing the electrical parameters of silicon through the use of neon ion implantation have resulted in the development of the authorial methodology for the generation and identification of additional levels of energy in the silicon band structure, improving the efficiency of photovoltaic cells made based on it [88].

The research has been directed at determining the effect of the degree and type of silicon defect in terms of the possibility of producing intermediate energy levels in the semiconductor’s band gap, thereby increasing the efficiency of solar cells by enabling a multi-step transition of electrons from the valence band to the intermediate band and then to the conduction band.

The object of our research is a method of producing intermediate energy levels in the band gap of n- and p-type silicon, with a specific resistivity *ρ* ranging from 0.25 Ω·cm to 10 Ω·cm, by generating deep radiation defects in the crystal structure of the semiconductor by implantation of Ne^+^ neon ions. The research material is doped with elements such as boron, phosphorus, and antimony.

Neon ions were chosen because the ions primarily produce point defects, the deliberate introduction of which into the crystalline lattice of silicon in the process of implantation makes it possible to alter its fundamental electrical parameters, including energy gap width and resistivity. The parameters significantly affect internal losses in photovoltaic cells [89]. Experimental studies were conducted to provide details for determination of the optimal dose of implanted neon ions because of their ability to generate intermediate energy levels in the semiconductor band gap.

### The Results of the Author’s Research

**Figure 19:**
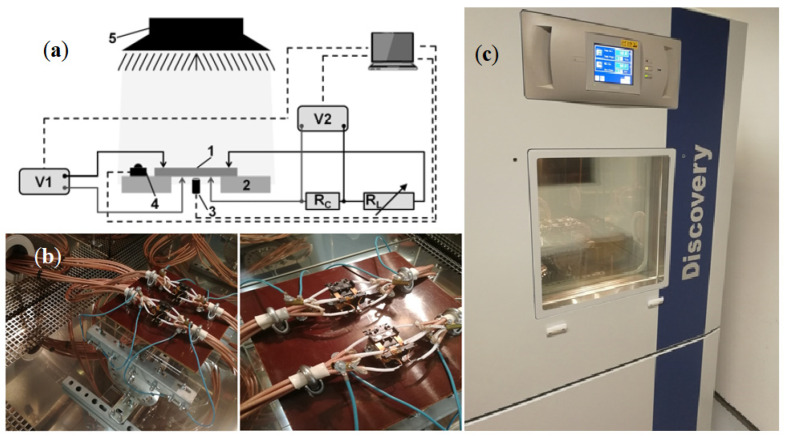
Silicon samples laboratory stand. (**a**) Schematic diagram of the laboratory stand: 1—solar cell, 2—supporting construction, 3—temperature sensor, 4—pyranometer, 5—light source, V1—Fluke 289, V2—The LCR-8110G Series LCR meter, RC—shunt resistor, RL—adjustable load. (**b**) Special measuring holders inside the climate chamber to hold silicon samples. (**c**) Discovery DY600C climate chamber [90].

The silicon samples were implanted with neon ions of energy *E* = 100 keV and different doses *D* using a UNIMAS 79 ion implanter and then isochronically annealed at 598 K for 15 min in a resistance furnace. The electrical parameters of the silicon samples were tested using a Discovery DY600C climate chamber using the proprietary PV Cells Meter computer program and the Winkratos software. A GW Instek LCR-8110G Series LCR meter was used to measure capacitance and conductance values, while sample temperature values were measured using Fluke 289 and Lutron TM-917 multimeters (Figure 19).

**Figure 20:**
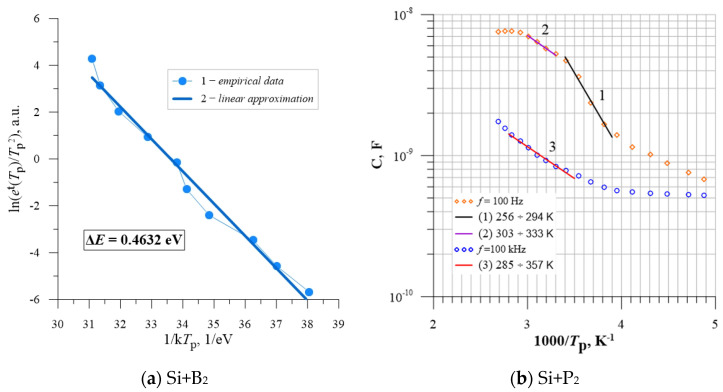
The Arrhenius law approximation ranges for silicon implanted with neon Ne^+^ ions of energy *E* = 100 keV (**a**) P-type silicon doped with boron, *ρ* = 0.4 Ω·cm, *D* = 2.2 × 10^14^ cm^−2^, Δ*E* = 0.46 eV. (**b**) N-type silicon doped with phosphorus, *ρ* = 10 Ω·cm, *D* = 4.0 × 10^14^ cm^−2^, Δ*E* = 0.23 eV [86,87].

The resulting capacitance and conductance measurements allowed us to determine the position values of the additional energy levels in the band gap. Two methods were used for this purpose. The first is the Thermal Admittance Spectroscopy (TAS) method, by which it was possible to determine the *e*^t^(*T*_p_) rate that determines the thermal emission, followed by the Arrhenius curves. By using the Arrhenius equation, it was possible to determine the activation energies of the deep energy levels by approximating the experimental data with a linear function [86]. An example of the results obtained by the TAS method is shown in Figure 20a.

Another method of determining the activation energy is the approximation of selected parts of the course *C*_p_ = f(1000/*T*_p_) with the function of the equation ln(y) = Ax + B, where *C*_p_ is the unit capacitance of the tested sample, and *T*_p_ is the temperature of the sample during the measurements performed at the frequency of the measuring signal *f* = 100 kHz. This in turn allowed the calculation of the conduction activation energy Δ*E*, which determines the depth of the additional intermediate energy level [87]. An example of the results obtained by the Arrhenius curve approximation method is shown in Figure 20b.

On the basis of the conducted research, it was possible to identify radiation defects that create additional energy levels in the silicon band gap, with corresponding activation energies, where the results are shown in Table 1. Our research proved that the implantation of Ne+ ions results in generating radiation defects in the crystal lattice of silicon as a photovoltaic cell base material and enables the generation of intermediate levels of energy in the band gap, improving the efficiency of photovoltaic cells made on its basis.

## 4. Conclusions

Solar energy is one of the most demanding renewable sources of electricity. Electricity production using photovoltaic technology not only helps meet the growing demand for energy, but also contributes to mitigating global climate change by reducing dependence on fossil fuels. The level of competitiveness of innovative next-generation solar cells is increasing due to the efforts of researchers and scientists related to the development of new materials, particularly nanomaterials and nanotechnology.

It is noted that the solar cell market is dominated by monocrystalline silicon cells due to their high efficiency. About two decades ago, the efficiency of crystalline silicon photovoltaic cells reached the 25% threshold at the laboratory scale. Despite technological advances since then, peak efficiency has now increased very slightly to 26.6%. As the efficiency of crystalline silicon technology approaches the saturation curve, researchers around the world are exploring alternative materials and manufacturing processes to further increase this efficiency. Polycrystalline and amorphous thin film silicon cells are seen as a serious competitor to monocrystalline silicon cells. However, their disadvantage is their disordered nature which results in low efficiency.

In this paper is a comprehensive overview of various PV technologies that are currently available or will be available in the near future on a commercial scale. A comparative analysis in terms of efficiency and the technological processes used is presented. Over the past few decades, many new materials have emerged that provide an efficient source of power generation to meet future demands while being cost-effective. This paper is a comprehensive study covering the generations of photovoltaic cells and the properties that characterize these cells. Photovoltaic cell materials of different generations have been compared based on their fabrication methods, properties, and photoelectric conversion efficiency.

First-generation solar cells are conventional and based on silicon wafers. The second generation of solar cells involves thin film technologies. The third generation of solar cells includes new technologies, including solar cells made of organic materials, cells made of perovskites, dye-sensitized cells, quantum dot cells, or multi-junction cells. With advances in technology, the drawbacks of previous generations have been eliminated in fourth-generation graphene-based solar cells. The popularity of photovoltaics depends on three aspects—cost, raw material availability, and efficiency. Third-generation solar cells are the latest and most promising technology in photovoltaics. Research on these is still in progress. This review pays special attention to the new generation of solar cells: multi-junction cells and photovoltaic cells with an additional intermediate band.

Recent advances in multi-junction solar cells based on n-type silicon and functional nanomaterials such as graphene offer a promising alternative to low-cost, high-efficiency cells. Currently, multi-junction cells, which benefit from advances enabled by nanotechnology, are breaking efficiency records. They are still quite expensive and represent a complex system, but there are simpler alternatives that may eventually provide a path to the competitiveness of the highest efficiency devices. Another significant advance is being made in the generation of additional energy levels in the band structure of silicon. In both cases, more research evidence, policies, and technology are needed to make them accessible. Therefore, it remains crucial to develop silicon-based technologies. The use of these new solar cell architectures would provide a new direction toward achieving commercial goals. Multi-junction based solar cells and new photovoltaic cells with an additional intermediate energy level are expected to provide extremely high efficiency. The research in this case focuses on a low-cost manufacturing process. Therefore, commercialization of these cells requires further work and exploration.

Nanotechnology and newly developed multifunctional nanomaterials can help overcome current performance barriers and significantly improve solar energy generation and conversion through photovoltaic techniques. Many physical phenomena have been identified at the nanoscale that can improve solar energy generation and conversion. However, the challenges associated with these technologies continue to be an issue when they are incorporated into PV manufacturing. Thanks to initial successes in recent years, nanomaterials are one of the most promising energy technologies of the future and are expected to significantly reform the future energy market. Carbon nanoparticles and their allotropic forms, such as graphene, are expected to offer high efficiency compared to conventional silicon cells in the near future and thus contribute to new prospects for the solar energy market.

## Figures and Tables

**Table 1:** Determination of intermediate energy levels for boron and phosphorus doped silicon samples implanted with Ne^+^ ions and energy *E* = 100 keV, isochronically annealed at 598 K [86,87].

Sample	Label	Resistivity	Dose	Activation Energy
**Silicon solar cells (p-type)** **doped with boron**	Si+B_1_	*ρ* = 0.4 Ω·cm	*D* = 4.0 × 10^13^ cm^−2^	Δ*E*_1_ = 0.34 eV
**Silicon solar cells (p-type)** **doped with boron**	Si+B_2_	*ρ* = 0.4 Ω·cm	*D* = 2.2 × 10^14^ cm^−2^	Δ*E*_2_ = 0.46 eV
**Silicon solar cells (p-type)** **doped with boron**	Si+B_3_	*ρ* = 0.4 Ω·cm	*D* = 4.0 × 10^14^ cm^−2^	Δ*E*_3_ = 0.32 eV
**Silicon solar cells (n-type)** **doped with phosphorus**	Si+P_1_	*ρ* = 10 Ω·cm	*D* = 4.0 × 10^13^ cm^−2^	Δ*E*_4_ = 0.19 eV
**Silicon solar cells (n-type)** **doped with phosphorus**	Si+P_2_	*ρ* = 10 Ω·cm	*D* = 4.0 × 10^14^ cm^−2^	Δ*E*_5_ = 0.23 eV

## Data Availability

Not applicable.
